# Enhanced Spatial Fuzzy C-Means Algorithm for Brain Tissue Segmentation in T1 Images

**DOI:** 10.1007/s12021-024-09661-x

**Published:** 2024-04-24

**Authors:** Bahram Jafrasteh, Manuel Lubián-Gutiérrez, Simón Pedro Lubián-López, Isabel Benavente-Fernández

**Affiliations:** 1 Biomedical Research and Innovation Institute of Cádiz (INiBICA) Research Unit, Puerta del Mar University Hospital, Cádiz, 11008 Spain; 2 Division of Neonatology, Department of Paediatrics, Puerta del Mar University Hospital, Cádiz, 11008 Spain; 3 Area of Paediatrics, Department of Child and Mother Health and Radiology, Medical School, University of Cádiz, Cádiz, 11003 Spain

**Keywords:** Magnetic resonance imaging (MRI), Brain tissue segmentation, Enhanced spatial fuzzy C-means (esFCM) algorithm, 3D T1 MRI images, Structural similarity index (SSIM)

## Abstract

Magnetic Resonance Imaging (MRI) plays an important role in neurology, particularly in the precise segmentation of brain tissues. Accurate segmentation is crucial for diagnosing brain injuries and neurodegenerative conditions. We introduce an Enhanced Spatial Fuzzy C-means (esFCM) algorithm for 3D T1 MRI segmentation to three tissues, i.e. White Matter (WM), Gray Matter (GM), and Cerebrospinal Fluid (CSF). The esFCM employs a weighted least square algorithm utilizing the Structural Similarity Index (SSIM) for polynomial bias field correction. It also takes advantage of the information from the membership function of the last iteration to compute neighborhood impact. This strategic refinement enhances the algorithm’s adaptability to complex image structures, effectively addressing challenges such as intensity irregularities and contributing to heightened segmentation accuracy. We compare the segmentation accuracy of esFCM against four variants of FCM, Gaussian Mixture Model (GMM) and FSL and ANTs algorithms using four various dataset, employing three measurement criteria. Comparative assessments underscore esFCM’s superior performance, particularly in scenarios involving added noise and bias fields.The obtained results emphasize the significant potential of the proposed method in the segmentation of MRI images.

## Introduction

Magnetic Resonance Imaging (MRI) of the human brain stands as one of the most important tools in neurological diagnosis, facilitating the identification of abnormalities and assessment of brain functionality. The segmentation of MRI data, involving the partitioning of the image into distinct regions with similar intensity, texture, and homogeneity, emerges as a critical task in the realm of medical image analysis. This segmentation extends to differentiating various brain tissues, such as White Matter (WM), Gray Matter (GM), and Cerebrospinal Fluid (CSF), playing a key role in the quantitative analysis of the brain for diagnostic purposes and further subdivision of brain regions. The precision achieved through accurate tissue segmentation and lesion separation enhances the ability of medical professionals to diagnose brain injury, neurodegenerative diseases like Alzheimer’s and Parkinson’s among others (Yang et al., 2008; Adhikari et al., 2015).

In numerous scenarios, the localization of pathology and accurate diagnosis is nearly impossible without the prior segmentation of brain tissues, highlighting the vital role of this process in the medical field. Brain tissue segmentation significantly contributes to the improved diagnosis of central nervous system diseases, constituting a major challenge in medical image analysis due to the inherent variations in images arising from different modalities, signal intensities, and device configurations. Factors such as differences in size, texture, shape, and unclear boundaries between regions compound the complexity of the segmentation problem.

Numerous approaches have been proposed for brain tissue segmentation (Kumar et al., 2018; Kalavathi, 2013; Al-Dmour & Al-Ani, 2018). These algorithms can be broadly categorized into two classes: manual-based and non-manual-based algorithms. Manual segmentation, relying on experts with extensive knowledge of radiology and neurology, necessitates proper segmentation tools and a significant amount of time. However, it also introduces subjectivity and inter-expert variability, requiring further decision-making processes Dora et al. (2017).

Non-manual-based algorithms encompass methods based on thresholding Otsu (1979), supervised learning approaches including deep learning Kumar et al. (2018), and clustering approaches. Thresholding methods are susceptible to the defined threshold, demanding considerable computational time to establish an appropriate threshold. Multilevel thresholding methods Gao et al. (2009), often combined with evolutionary algorithms, have been proposed to mitigate computational demands. Nevertheless, the accuracy of these methods depends on the image histogram and the presence of noise and artifacts in images.

Recently, supervised deep learning approaches (Moeskops et al., 2016; Brebisson & Montana, 2015; Zhang et al., 2015; Ding et al., 2020; Díaz-Pernas et al., 2021; Hua et al., 2021) have gained attention due to their learning ability. These methods learn from a set of images and generalize their performance to previously unseen images. However, their efficacy relies on a substantial amount of ground truth segmentation to comprehend the data distribution. Performance degradation occurs when the distribution of the input image deviates significantly from the training set. Ongoing efforts are underway to enhance their performance for diverse image datasets and modalities Hoopes et al. (2022).

In clustering approaches, pixels are grouped based on criteria such as connectivity, distance, and pixel intensity. The Gaussian Mixture Model (GMM) is a popular method in neuroimaging that employs Gaussian probability functions to cluster pixels/voxels in an image based on their intensity values, determined through an Expectation Maximization (EM) algorithm. However, most clustering approaches, including GMM, face challenges in easily segmenting images with intensity inhomogeneity, commonly known as the bias field problem. To address this issue, bias field correction methods are employed before or during segmentation Liu and Zhang (2013). Maximum Likelihood (ML)-based GMM methods may encounter issues of overfitting or getting trapped in local minima Dora et al. (2017). An alternative method is Maximum A Posteriori (MAP) estimation Zhang et al. (2001). Combining GMM with Markov random field, Markov Chain Monte Carlo, and evolutionary algorithms has been explored to improve segmentation performance (Zhang et al., 2014; Saladi & Amutha Prabha, (2018).

Fuzzy C-means (FCM) is one of the most popular approaches that utilizes a similarity criterion to segment brain tissues (Ji et al., 2011; Chuang et al., 2006; Mahata & Sing, 2020; Maitra et al., 2019; Singh & Bala, 2021; Li et al., 2014; Chahal & Pandey, 2023). A membership function assigns a voxel to a class based on a degree determined by a membership function. However, FCM is less suitable for noisy images and in the presence of intensity inhomogeneity. Preprocessing steps are sometimes implemented before using FCM to mitigate these challenges. Modified FCMs with bias field correction, designed to be robust against noise, have been proposed to overcome these issues (Ji et al., 2011; Chuang et al., 2006; Mahata & Sing, 2020; Maitra et al., 2019; Singh & Bala, 2021; Li et al., 2014). Notably, many of these proposed approaches have been tailored for 2D MRI images, potentially limiting their applicability to 3D images or synthetic MRI images, which may differ from real images.

In this study, we introduce an Enhanced Spatial Fuzzy C-means (esFCM) algorithm with simultaneous bias correction to enhance the segmentation of 3D T1 MRI images. The esFCM employs a weighted least-squares method for bias field measurement, utilizing weights derived from the structural similarity index (SSIM) and neighborhood pixel information from the previous iteration to refine the FCM algorithm. This algorithmic enhancement aims to address the challenges posed by intensity inhomogeneity and contribute to more accurate and reliable segmentation results.

## Related Work

The introduction of a spatial Fuzzy C-means (sFCM) emphasizes the role of neighboring pixels in membership degree assignment, incorporating a spatial function into the membership function to consider the influence of neighboring pixels in the images Chuang et al. (2006). Expanding on this idea, Adhikari et al. (2015) introduces a conditional version of sFCM that considers the involvement level of pixels in the final membership function by incorporating information from neighboring pixels. This adaptation aims to enhance the adaptability of the algorithm to complex image structures and varying pixel relationships.

Sikka et al. (2009) shift the focus to image quality enhancement through a sophisticated filter based on discrete Fourier transformations, strategically employed to remove bias fields and manipulate the end-of-image histogram for effective contrast stretching. Cluster centers are estimated based on specific criteria derived from the image histogram, and a modified FCM method is applied for image segmentation. Notably, post-processing methods are then employed to discern and separate ambiguous pixels from the rest, reflecting an emphasis on refining segmentation outcomes.

An approach combines a probabilistic partition matrix that constrains the membership function with an energy minimization method grounded in coherent local intensity clustering proposed in Ji et al. (2011). This method introduces a refined balance between local and global intensity considerations within the image. In the work conducted by Maitra et al. (2019), the proposal involves a combination of local and global membership functions to address image inhomogeneity and noise. The local membership function is intricately computed through a normalized multiplication of two metrics: a distance metric and a binary metric. The distance metric encapsulates neighborhood intensity distances concerning cluster centers, while the binary metric assigns one for minimum intensity distances and zero otherwise, contributing to a comprehensive segmentation strategy.

More recently, Mahata and Sing (2020) presented a modified FCM algorithm featuring local and global membership functions constrained by local spatial information. A regularizer is introduced to strike a balance between global and local factors, thereby refining segmentation outcomes. The proposed spatially constrained likelihood-based local entropy (FCMGsLE) is validated using simulated and real 3D human brain datasets.

Pre-filtering images using a local Zernike moment-based unbiased nonlocal means approach, strategically designed to eliminate noise before addressing the bias field proposed in Singh and Bala (2021). This method utilizes M orthogonal polynomials of degree P for bias field removal, with the degree of polynomials, filtering parameter, and order of moments empirically determined, underscoring the nuanced influence of these parameters on segmentation results.

A combination of dictionary learning and improved FCM is proposed to mitigate the impact of noise in MRI image segmentation Miao et al. (2020). However, it is acknowledged that this algorithm may lack robustness concerning intensity inhomogeneity, signifying an avenue for potential improvement. Tavakoli-Zaniani et al. (2021) modified the FCM’s objective function and adopted a double estimation approach for image segmentation, employing both the original and denoised images as inputs. The challenge of accurately inferring and removing noise from the image before applying the FCM method is highlighted. An integration of probabilistic intuitionistic fuzzy set theory and spatial neighborhood information porposed for MRI segmentation by Solanki and Kumar (2023). However, the approach’s reliance on an extensive grid search to find optimal parameters is acknowledged as a practical limitation.

## The Proposed esFCM

In this section, we present the proposed enhanced spatial fuzzy C-means (esFCM) for MRI segmentation, introducing novel methodologies inspired by the structural similarity index (SSSIM) to mitigate the impact of bias field and spatial information to reduce the noise impact in brain tissue segmentation.

Automated segmentation of brain tissues in MRI images demands consideration of inhomogeneity, noise, and the spatial relationships between pixels. While Fuzzy C-means (FCM) stands as a conventional and commonly used intensity-based clustering approach for image segmentation, its conventional algorithms struggle with noise and the impact of neighboring pixels during segmentation. To address this, a spatial FCM (sFCM) was introduced in Chuang et al. (2006), designed for 2D images without accounting for bias field correction. Motivated by sFCM, our enhanced approach, esFCM, considers the relationships among neighborhood pixels and aims to reduce noise impact in 3D MRI images.

Considering a 3D image $\textbf{I}$ of size $H \times W \times D$ and a filtered image $\hat{\textbf{I}}$, we perform segmentation into *C* individual clusters, each represented by a center $V_{i}$. Assigning a degree of membership to each voxel in the images via the membership function $\mu _{i}$, where each voxel belongs to a cluster with a specific confidence degree, is expressed by

1 $$\begin{aligned} \mu _{ij} = \frac{1}{\sum _{c=1}^{C} \left( \frac{D_{ij} }{D_{cj}} \right) ^{\frac{2}{m-1}} } \end{aligned}$$

where $D_{ij}$ is the distance between *j*th voxel in the image and the *i*th cluster center

2 $$\begin{aligned} D_{ij} = || \hat{I}_j - V_i || \end{aligned}$$

ensuring that the sum of membership function values for each voxel is one

3 $$\begin{aligned} \sum _{i=1}^{C} \mu _{ij} = 1 \end{aligned}$$

Then, the cluster centers are updated using

4 $$\begin{aligned} V_i = \frac{ \sum _{j=1}^{n} \mu _{ij}^{m} I_{j} }{\sum _{j=1}^{n} \mu _{ij}^{m}} \end{aligned}$$

where *n* is the total number of voxels in a 3D image.

Here, we introduce an energy function ($E_{ij}$) to consider the impact of neighboring pixels.

5 $$\begin{aligned} E_{ij} = \frac{ \sum _{j=1}^{P} \bar{\mu }_{ij}^{t-1} }{P} \end{aligned}$$

where *P* is the number of neighbourhood pixel around each voxel, here p=6 and $\bar{\mu }_{ij}^{t-1}$ is the membership value obtained from the previous iteration. Then, the final membership function obtained by the pointwise multiplication of the initial membership function and $E_{ij}$:

6 $$\begin{aligned} \bar{\mu }_{ij} = \mu _ij \odot E_{ij} \end{aligned}$$

where $\odot$ stands for the point wise multiplications. Now we address the inhomogeneity. A common assumption is that the inhomogenity in an image is a smooth, low-frequency intensity variation across the image. It can often be effectively modeled as a polynomial function of low degree. A least squares equation can be used to reduce the inhomogeneity (bias filed) in a given image as follows

7 $$\begin{aligned} \hat{\beta } = (A^T A)^{-1} A^T (\textbf{I} - \textbf{L}) \end{aligned}$$

where matrix *A* represents a quadratic polynomial function of the image coordinates in reference space, and $\textbf{L}$ is the prediction of the original image $\textbf{I}$. In the context of the FCM framework, the utilization of cluster centers to predict the value of the *j*-th voxel in $\textbf{L}$ is expressed by the equation:

8 $$\begin{aligned} L_j = \sum _{i=1}^{C}{V_i \cdot \mu _{ij}} \end{aligned}$$

While the conventional least square Eq. 7 for bias field removal treats every point in the images with equal weight, this global approach may not be optimal, especially considering the local impact of bias fields. To address this limitation, we propose a weighted version of the least square equation. Specifically, we compute the Structural Similarity Index (SSIM) map between the image gradient and the predicted image $\textbf{L}$, enhancing the emphasis on borders among different crucial regions in the images.

The SSIM, introduced by Wang et al. (2004) for measuring image similarity, is computed as follows to preserve details while highlighting contrast differences in the gradient of real image $\mathbf {I_g}$ and the gradient of predicted image $\mathbf {L_g}$:

9 $$\begin{aligned} SSIM(\mathbf {I_g}, \mathbf {L_g}) = \frac{{ (2\Lambda _{I_g}\Lambda _{L_g} + c_1) (2\Sigma _{I_{g}L_{g}} + c_2) }}{{ (\Lambda _{I_g}^2 + \Lambda _{L_g}^2 + c_1) (\Sigma _{I_g}^2 + \Sigma _{L_g}^2 + c_2) }} \end{aligned}$$

Here, constants $c_1 = 0.0001$ and $c_2=0.0009$, $\Lambda$ denotes the mean intensity, and $\Sigma$ represents the standard deviation. The SSIM map highlights regions that should be given more importance and/or have been neglected.

After computing SSIM, the weighted least square equation is employed to determine the required weights for bias field correction:

$$\begin{aligned} \hat{\beta } = (A^T W A)^{-1} A^T W (\textbf{I} - \textbf{L}) \end{aligned}$$

Here, matrix *A* is the quadratic polynomial function of the image coordinates, and *W* denotes the weights derived from the SSIM computation. Subsequently, the filtered image $\hat{\textbf{I}}$ is obtained through Eq. 10:

10 $$\begin{aligned} \hat{\textbf{I}} = \textbf{I} - \hat{\beta } A \end{aligned}$$

Algorithm 1 outlines the procedure of esFCM for segmenting a 3D image. Additionally, the average SSIM value is utilized as a cost function, aiding in the selection of the best-filtered image based on its similarity to the original image.

**Algorithm 1:**
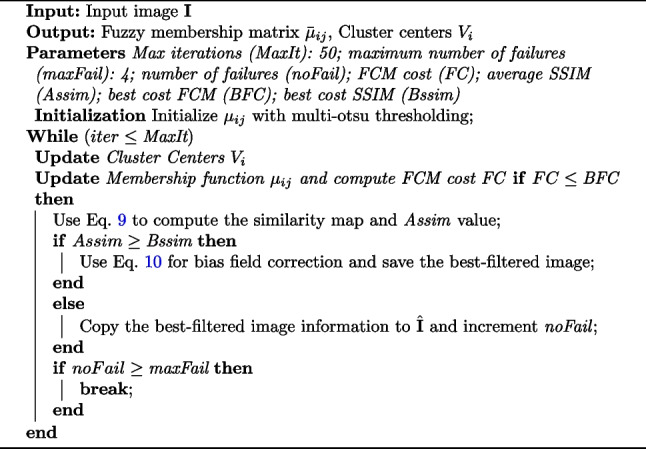
Pseudo Code of the Proposed esFCM Algorithm

## Experimental Setup

We assess the effectiveness of the proposed algorithm through extensive evaluations on datasets derived from four distinct studies:

- The BrainWeb dataset (Cocosco, 1997; Kwan et al., 1996, 1999; Collins et al., 1998) comprises 20 simulated T1 MRI images along with corresponding ground truth. All images have uniform spacing set at 1*mm*. The image dimensions are $181 \times 256 \times 256$. The ground truth segmentation has dimensions of $362 \times 434 \times 362$ with a spacing of 0.5*mm*. We employ the SimpleITK library to adjust the size and spacing of the segmentation to match that of the image size.
- The Internet Brain Segmentation Repository (IBSR) consists of 18 T1 MRI images accompanied by their respective ground truth segmentations. Similar to the BrainWeb dataset, the IBSR images exhibit specific size and spacing characteristics. Both the image and segmentation dimensions for the IBSR dataset are $256 \times 128 \times 256$, and they feature variable spacing, specifically 0.94, 1.5, and 0.94 mm, respectively.
- Three T1 MRI images from eight-year-old preterm born patients acquired at Puerta del Mar University Hospital in Cádiz, Spain, and segmented using MELAGE software Jafrasteh et al. (2023) by medical experts in the group, referred as HUPM dataset https://rodin.uca.es/handle/10498/25580. The brain extraction is done using the deep learning tools inside MELAGE. The image spacing is equal to 1*mm*. The image size is $192 \times 256 \times 144$.
- We included 581 T1-weighted (T1w) images from the IXI dataset^1^ The voxel spacing in each dimension is 0.94 mm x 0.94 mm x 1.5 mm.

To gauge the robustness of the proposed algorithm, we introduced artificial noise (0%, 20%, and 40%) and bias field (0%, 5%, and 10%) to the images from all datasets. The comparison involved a 369 3D augmented images to be segmented by different algorithms in this study. This facilitated a comprehensive performance comparison with other algorithms employed in this study. The performance of the proposed esFCM is benchmarked against FCM, FCMGsLE Mahata and Sing (2020), spFCM Maitra et al. (2019), and sFCM Chuang et al. (2006). All algorithms were initialized using the multiOtsu thresholding algorithm Liao et al. (2001), with a maximum iteration limit set to 50. Moreover, we included Gaussian Mixture Model (GMM) Greenspan et al. (2006) , FSL fast segmentation Zhang et al. (2001) and ANTs(atropos) Avants et al. (2011) in the comparison. Since there is no ground truth for IXI datasets, in this dataset, we compare the performance of the proposed esFCM against GMM, ANTS, and FSL, where the proposed method is considered the ground truth. This study can provide valuable insights into its difference in accuracy, robustness, and computational efficiency compared to established segmentation algorithms in the absence of a definitive reference standard. By evaluating its performance against alternative methods under these conditions, we can better understand the relative difference among these algorithm, thus contributing to the broader understanding of applicability of esFCM and its potential in the MRI image segmentation task.

Evaluation metrics include:

- Hausdorff distance (HD) between the ground truth and segmented image.
  11 $$\begin{aligned} \text {Hausdorff Distance} = \max \left( \max _{p \in \text {G}} \min _{q \in \text {S}} ||p - q||, \max _{q \in \text {S}} \min _{p \in \text {G}} ||q - p|| \right) \end{aligned}$$
  Where G stands for ground truth and S represents the segmented image using variants of FCMs.
- DICE similarity measure:
  12 $$\begin{aligned} \text {DICE} = \frac{2 \times \text {TP}}{(\text {TP}+\text {FN}) + (\text {TP} + \text {FP})} \end{aligned}$$
  where TP denotes True Positive, FN signifies False Negative, and FP represents False Positive.
- Segmentation accuracy: the percentage of agreement between the ground truth and the segmented image.
  13 $$\begin{aligned} \text {Accuracy} = \frac{\text {TP} + \text {TN}}{\text {TP} + \text {TN} + \text {FP} + \text {FN}} \end{aligned}$$
  where FN stands for False Negative.

Three types of tissue are segmented : 1) Cerebrospinal fluid (CSF), 2) White matter (WM), and 3) Gray matter (GM).

## Results and Discussion

Tables 1, 2, 3 and 4 provide a comprehensive comparison of various algorithms in our study based on the DICE similarity measure. The findings highlight the impact of adding a bias field on the segmentation performance of all the methods under investigation.

It is evident from the results that the introduction of a bias field or noise can lead to a reduction in segmentation accuracy for most methods. Notably, our proposed algorithm demonstrates superior segmentation accuracy even under these challenging conditions. Specifically, in the absence of additional bias field but with a 20 percent addition of noise, the performance of spFCM and esFCM for CSF segmentation becomes very close. However, esFCM maintains better accuracy for WM and GM segmentation.

**Fig. 1:**
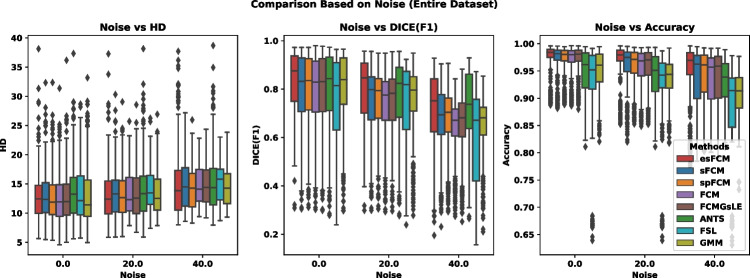
Comparison of various algorithms employed in this study in terms of noise amount, evaluated based on the HD, DICE, and Accuracy criteria from left to right

**Fig. 2:**
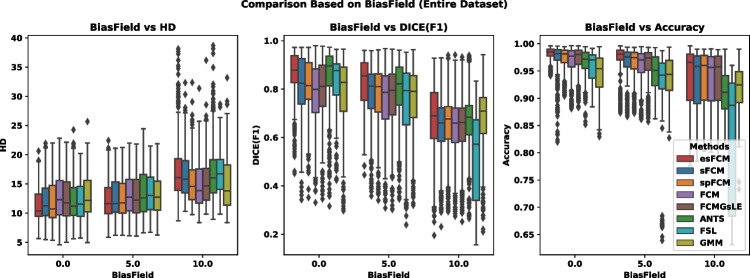
Comparison of various algorithms employed in this study in terms of bias field amount, evaluated based on the HD, DICE, and Accuracy criteria from left to right

Figures 1 and 2 further illustrate algorithmic comparisons based on the amount of added noise and bias field in the images, respectively, using criteria such as Hausdorff Distance (HD), DICE, and Accuracy. It is noteworthy that the FCM algorithm exhibits a pronounced reduction in DICE similarity measure with increasing noise and bias field, underscoring its sensitivity to these factors. In contrast, our proposed esFCM algorithm consistently outperforms other methods across all criteria, demonstrating its robustness.

**Fig. 3:**
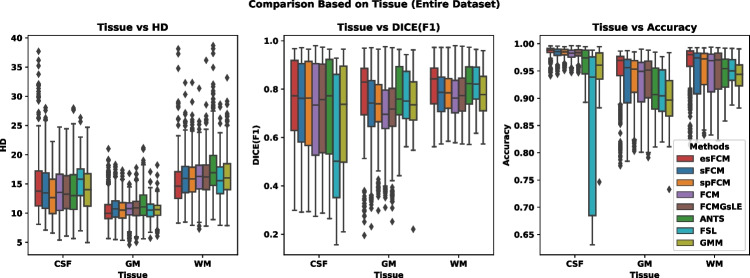
Comparison of various algorithms employed in this study in terms of three different tissues, evaluated based on the HD, DICE, and Accuracy criteria from left to right

Figure 3 presents the specific results for three tissue types-CSF, GM, and WM-with varying amounts of added bias field and noise. The segmentation of CSF emerges as particularly challenging, exhibiting lower accuracy across the algorithms. Notably, esFCM proves comparable to other algorithms in terms of DICE similarity measure, HD, and accuracy for CSF segmentation, while outperforming them for GM and WM tissue segmentation. FSL algorithm shows high standard deviation regarding accuracy for CSF segmetnation, which is related to its sensitivity to the additional bias filed into the image.

**Fig. 4:**
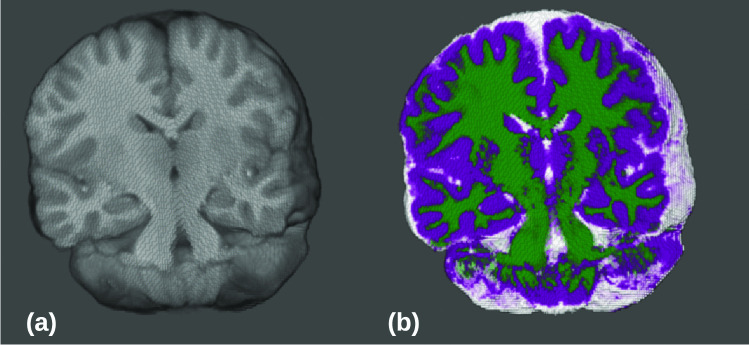
The image depicts a selected 3D region (**a**) and the corresponding segmentation using esFCM (**b**) from a preterm-born eight-year-old patient in the HUPM dataset. In the visualization, cerebrospinal fluid (CSF) is represented in white, white matter (WM) in green, and gray matter (GM) in purple. 3D visualization using MELAGE software

Figure 4 shows a 3D visulaization of one of an MRI image from HUPM dataset, with the corresponding segmentation using the proposed esFCM algorithm. In summary, our results indicate that the proposed esFCM algorithm consistently outperforms other methods, displaying better accuracy and lower standard deviation across all criteria. It is essential to emphasize that the efficacy of esFCM is particularly pronounced when the proposed bias field correction is employed, preventing it from falling into the performance category of sFCM in its absence. This reinforces the importance of our novel bias field correction strategy in enhancing the overall performance of the esFCM algorithm. Table 2 provides a comprehensive comparison of segmentation performance across different tissue types using three segmentation algorithms, GMM, ANTS, and FSL. The ground truth is based on the segmentation results of esFCM, highlights algorithmic performance deviations. Overall, ANTs and GMM show strong performance across metrics and tissues. The results of esFCM can be close to GMM in terms of DICE coefficient. In terms of HD, FLS indicates more accurate segmentation in terms of spatial agreement. In terms of accuracy GMM and ANTs are more similar to the proposed esFCM.

The code for the proposed algorithm will be made publicly available upon publication. It is intended to be integrated into the MELAGE interactive segmentation platform.

**Table 1:** DICE similarity coefficient for all the datasets taken in this study with 0 percentage of added Gaussian noise and different amounts of bias field. The values in the parenthesis are the obtained standard deviation

Bias(%)	Tissue	spFCM	FCM	FCMGsLE	sFCM	esFCM	GMM	ANTS	FSL
0	CSF	0.78(0.21)	0.78(0.22)	0.78(0.22)	0.79(0.2)	**0.82(0.15)**	0.76(0.22)	0.77(0.21)	0.73(0.2)
	WM	0.91(0.05)	0.91(0.05)	0.92(0.05)	0.92(0.05)	**0.92(0.05)**	0.91(0.06)	0.92(0.05)	0.89(0.15)
	GM	0.88(0.08)	0.88(0.09)	0.88(0.09)	0.89(0.08)	**0.91(0.06)**	0.87(0.1)	0.88(0.09)	0.85(0.08)
5	CSF	0.77(0.21)	0.76(0.22)	0.76(0.22)	0.77(0.21)	**0.81(0.15)**	0.77(0.21)	0.76(0.22)	0.71(0.21)
	WM	0.88(0.05)	0.87(0.04)	0.87(0.05)	0.88(0.05)	**0.9(0.04)**	0.88(0.05)	0.88(0.05)	0.88(0.05)
	GM	0.85(0.08)	0.83(0.09)	0.84(0.09)	0.85(0.08)	**0.89(0.06)**	0.85(0.09)	0.85(0.09)	0.82(0.09)
10	CSF	0.72(0.22)	0.71(0.23)	0.71(0.22)	0.72(0.22)	**0.76(0.18)**	0.7(0.22)	0.71(0.24)	0.4(0.11)
	WM	0.74(0.04)	0.74(0.04)	0.74(0.04)	0.74(0.04)	**0.77(0.05)**	0.76(0.05)	0.74(0.05)	0.36(0.38)
	GM	0.69(0.05)	0.68(0.05)	0.68(0.05)	0.68(0.05)	**0.73(0.07)**	0.75(0.09)	0.69(0.06)	0.32(0.33)

**Table 2:** Comparison of segmentation performance on IXI datasets using DICE, HD, and Accuracy metrics

	Tissue	GMM	ANTS	FSL
DICE	CSF	0.85(0.07)	**0.87(0.1)**	0.84(0.07)
	WM	**0.94(0.04)**	0.9(0.21)	0.93(0.04)
	GM	**0.88(0.05)**	**0.88(0.12)**	0.86(0.05)
HD	CSF	13.17(2.81)	**9.77(3.22)**	12.76(2.51)
	WM	11.21(3.64)	13.7(14.03)	**10.71(4.14)**
	GM	6.95(1.23)	8.0(2.93)	**6.7(1.28)**
Accuracy	CSF	**0.98(0.01)**	**0.98(0.02)**	0.97(0.01)
	WM	**0.98(0.01)**	**0.98(0.03)**	**0.98(0.01)**
	GM	**0.96(0.02)**	**0.96(0.05)**	0.95(0.02)

**Table 3:** DICE similarity coefficient for all the datasets taken in this study with 20 percentage of added Gaussian noise and different amounts of bias field. The values in the parenthesis are the obtained standard deviation

Bias(%)	Tissue	spFCM	FCM	FCMGsLE	sFCM	esFCM	GMM	ANTS	FSL
0	CSF	0.8(0.16)	0.78(0.17)	0.78(0.19)	**0.81(0.14)**	**0.81(0.15)**	0.74(0.22)	0.77(0.2)	0.9(0.02)
	WM	0.83(0.05)	0.81(0.05)	0.83(0.05)	0.84(0.09)	**0.9(0.04)**	0.85(0.03)	**0.9(0.04)**	0.89(0.01)
	GM	0.82(0.06)	0.79(0.06)	0.81(0.06)	0.81(0.14)	**0.89(0.06)**	0.82(0.08)	0.87(0.08)	0.88(0.01)
5	CSF	0.75(0.21)	0.73(0.22)	0.75(0.21)	0.76(0.2)	**0.81(0.14)**	0.73(0.21)	0.75(0.21)	0.7(0.19)
	WM	0.82(0.03)	0.8(0.03)	0.81(0.03)	0.83(0.03)	**0.87(0.03)**	0.81(0.03)	0.84(0.03)	0.83(0.03)
	GM	0.79(0.05)	0.77(0.05)	0.78(0.05)	0.81(0.05)	**0.87(0.04)**	0.78(0.06)	0.82(0.07)	0.78(0.06)
10	CSF	0.68(0.2)	0.66(0.2)	0.67(0.2)	0.67(0.19)	**0.7(0.16)**	0.66(0.2)	0.68(0.22)	0.39(0.1)
	WM	0.71(0.04)	0.7(0.04)	0.71(0.04)	0.7(0.05)	**0.73(0.07)**	**0.73(0.04)**	0.72(0.04)	0.35(0.37)
	GM	0.62(0.06)	0.61(0.05)	0.61(0.06)	0.61(0.07)	0.66(0.11)	**0.7(0.06)**	0.66(0.05)	0.31(0.32)

**Table 4:** DICE similarity coefficient for all the datasets taken in this study with 40 percentage of added Gaussian noise and different amounts of bias field. The values in the parenthesis are the obtained standard deviation

Bias(%)	Tissue	spFCM	FCM	FCMGsLE	sFCM	esFCM	GMM	ANTS	FSL
0	CSF	0.76(0.15)	0.7(0.15)	0.72(0.17)	0.75(0.11)	0.78(0.15)	0.65(0.2)	0.76(0.19)	**0.85(0.02)**
	WM	0.78(0.04)	0.73(0.04)	0.76(0.05)	0.77(0.05)	0.84(0.02)	0.73(0.04)	**0.88(0.03)**	0.77(0.01)
	GM	0.75(0.03)	0.7(0.02)	0.72(0.04)	0.73(0.08)	0.85(0.03)	0.7(0.04)	**0.86(0.06)**	0.75(0.01)
5	CSF	0.69(0.19)	0.64(0.18)	0.67(0.18)	0.7(0.17)	**0.75(0.13)**	0.63(0.18)	0.72(0.2)	0.41(0.12)
	WM	0.74(0.04)	0.71(0.04)	0.72(0.05)	0.74(0.05)	**0.77(0.05)**	0.69(0.06)	**0.77(0.03)**	0.37(0.38)
	GM	0.68(0.04)	0.64(0.03)	0.65(0.05)	0.69(0.06)	**0.74(0.08)**	0.68(0.03)	**0.74(0.04)**	0.33(0.35)
10	CSF	**0.59(0.14)**	0.55(0.13)	0.55(0.12)	0.55(0.11)	0.55(0.1)	0.57(0.17)	0.58(0.15)	0.36(0.09)
	WM	0.65(0.05)	0.65(0.05)	0.65(0.05)	0.65(0.06)	0.66(0.08)	0.65(0.11)	**0.67(0.05)**	0.31(0.35)
	GM	0.51(0.12)	0.49(0.11)	0.48(0.14)	0.5(0.16)	0.51(0.21)	**0.59(0.07)**	0.57(0.08)	0.27(0.31)

## Conclusions

In this study, we present the Enhanced Spatial Fuzzy C-means (esFCM) algorithm designed for the segmentation of 3D T1 MRI images. The proposed algorithm integrates simultaneous bias field correction and incorporates innovative methodologies inspired by the Structural Similarity Index (SSIM) to address intensity inhomogeneity and reduce noise in brain tissue segmentation. Rigorous evaluations conducted on IBSR, Brain Web and HUPM dataset, attest to the robustness of esFCM. It demonstrates superior performance compared to other algorithms, especially in challenging conditions involving noise and bias field variations. Notably, the algorithm excels in the segmentation of specific tissue types, including Cerebrospinal Fluid (CSF), Gray Matter (GM), and White Matter (WM). The results emphasize the significance of the introduced bias field correction strategy in enhancing the overall accuracy and reliability of the esFCM algorithm. Additionally, we assessed the disparities between the segmentation outcomes of esFCM and those of GMM, ANTs, and FSL algorithms utilizing the IXI dataset. The bias field is getting more important in MRI segmentation of newborn and children. As a future direction, we plan to extend this work to encompass the segmentation of newborn pattients and also non-healthy MRI images, i.e.the ones contains lesion or tumor.

## Information Sharing Statement

The HUPM dataset used in this study received approval from the Research and Ethics Committee, and informed consent was obtained from all parents or guardians of the participants to use MRI data for research purposes.

## Figures and Tables

**Fig. 5:**
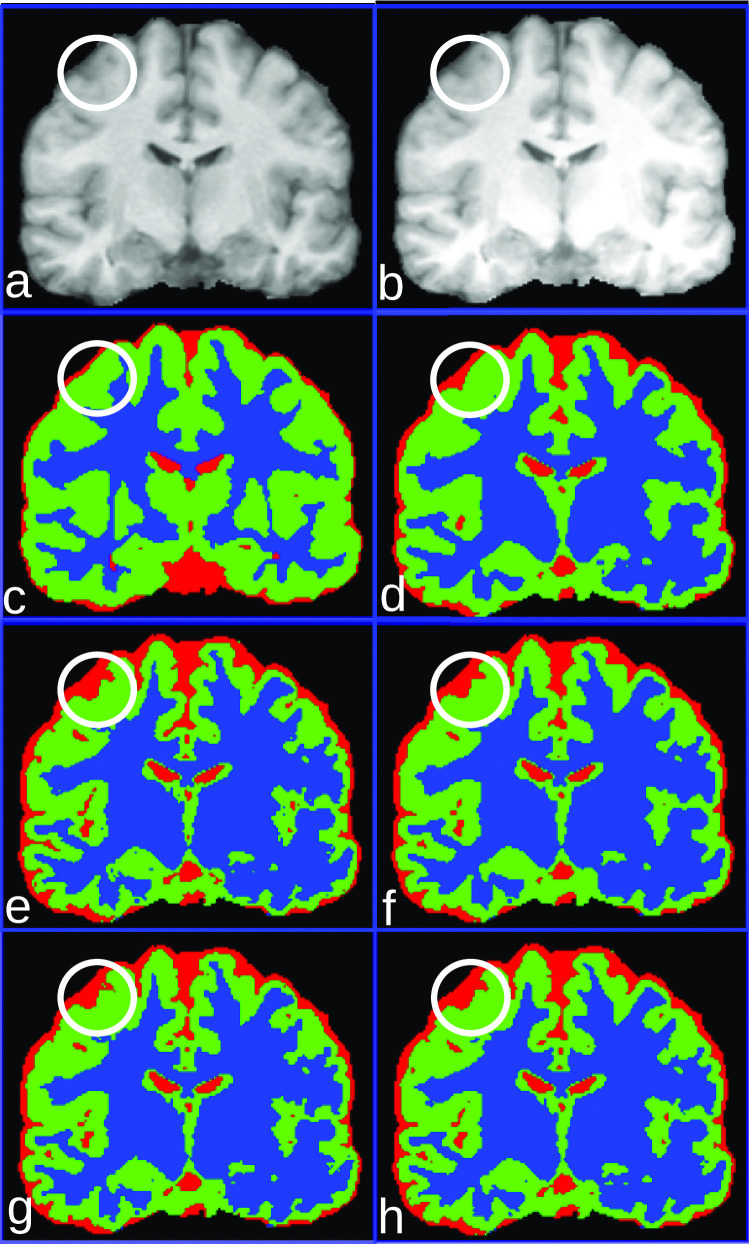
**a** A coronal section from an image obtained from IBSR dataset **b** the same image after adding 10 percent bias field. **c** ground truth segmentation **d** segmentation from the proposed algorithm **e** fcm **f** spFCM Maitra et al. (2019) **g** sFCM Chuang et al. (2006) and **h** FCMGsLE Mahata and Sing (2020)

**Fig. 6:**
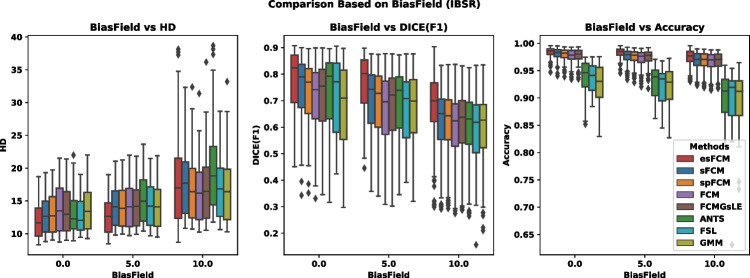
Comparison of various algorithms employed in this study in terms of bias field amount for IBSR dataset, evaluated based on the HD, DICE, and Accuracy criteria from left to right

**Fig. 7:**
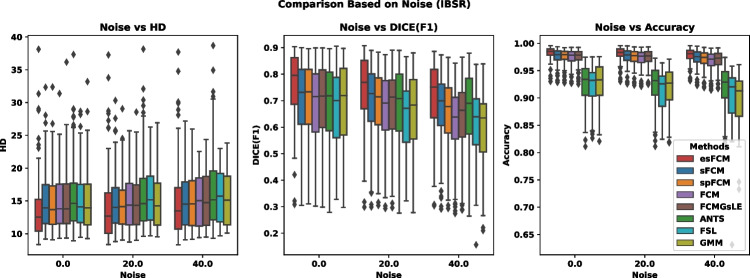
Comparison of various algorithms employed in this study in terms of noise amount for IBSR dataset, evaluated based on the HD, DICE, and Accuracy criteria from left to right

**Fig. 8:**
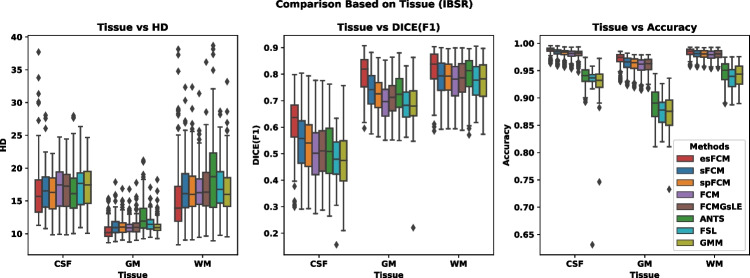
Comparison of various algorithms employed in this study in terms of three different tissues for IBSR dataset, evaluated based on the HD, DICE, and Accuracy criteria from left to right

**Fig. 9:**
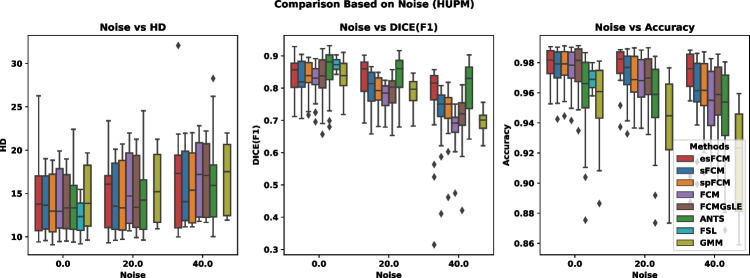
Comparison of various algorithms employed in this study in terms of noise amount for HUPM dataset, evaluated based on the HD, DICE, and Accuracy criteria from left to right

**Fig. 10:**
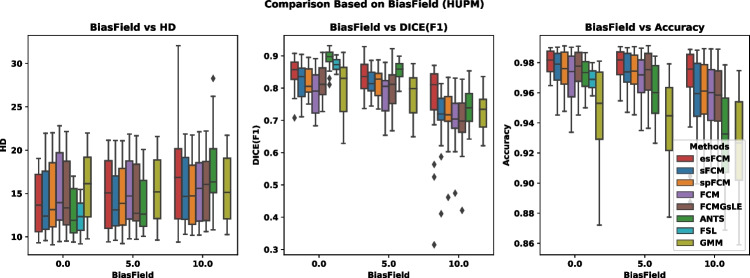
Comparison of various algorithms employed in this study in terms of bias field amount for HUPM dataset, evaluated based on the HD, DICE, and Accuracy criteria from left to right

**Fig. 11:**
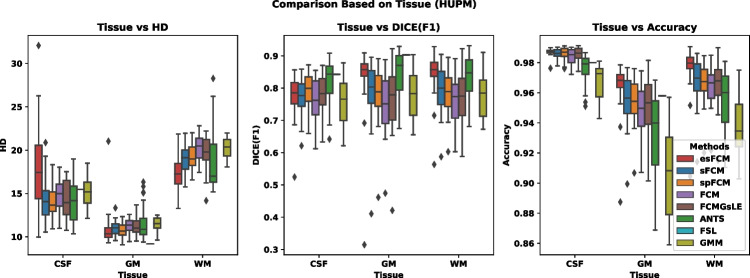
Comparison of various algorithms employed in this study in terms of three different tissues for HUPM dataset, evaluated based on the HD, DICE, and Accuracy criteria from left to right

**Fig. 12:**
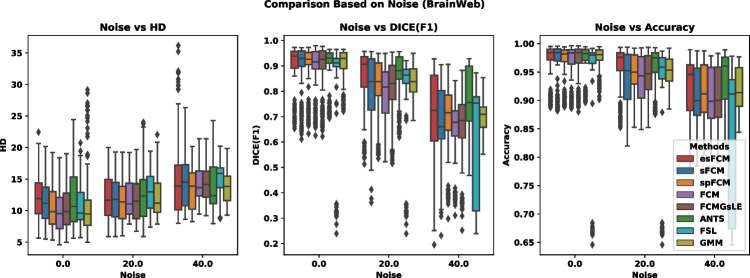
Comparison of various algorithms employed in this study in terms of noise amount for BrainWeb dataset, evaluated based on the HD, DICE, and Accuracy criteria from left to right

**Fig. 13:**
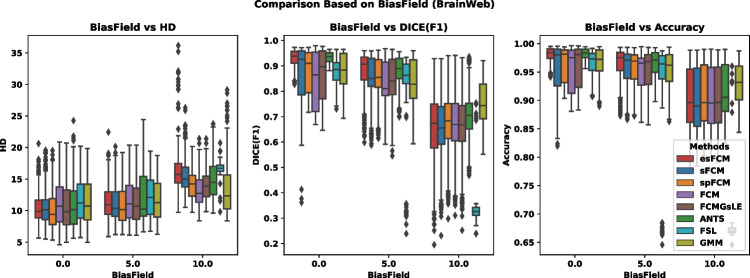
Comparison of various algorithms employed in this study in terms of bias field amount for BrainWeb dataset, evaluated based on the HD, DICE, and Accuracy criteria from left to right

**Fig. 14:**
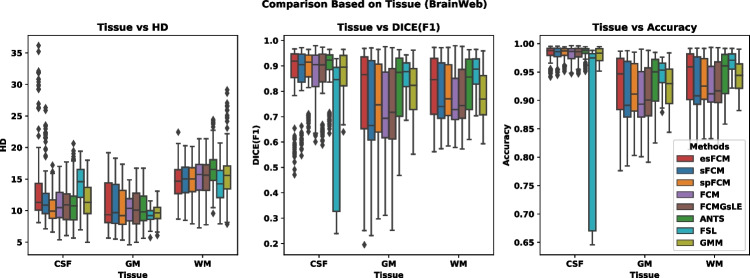
Comparison of various algorithms employed in this study in terms of three different tissues for BrainWeb dataset, evaluated based on the HD, DICE, and Accuracy criteria from left to right

**Fig. 15:**
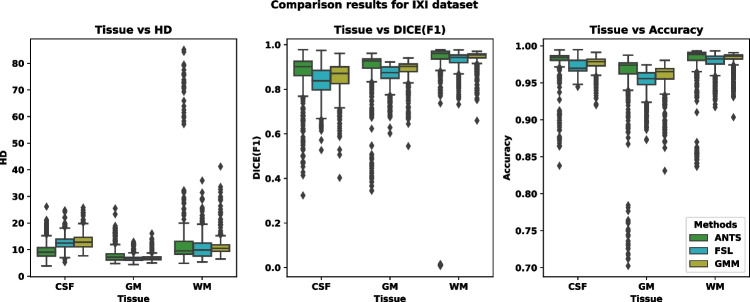
Comparison among GMM, ANTs and FSL algorithms employed in terms of three different tissues for IXI dataset, evaluated based on the HD, DICE, and Accuracy criteria from left to right

## Data Availability

The dataset used in this research is comprised of T1-weighted MRI images. It includes: IXI dataset: Available from http://brain-development.org/ixi-dataset. IBSR dataset: Available from https://www.nitrc.org/projects/ibsr. Brainweb dataset: Available from https://brainweb.bic.mni.mcgill.ca/brainweb/. HUPM dataset: Available from https://rodin.uca.es/handle/10498/31306.

## Appendix A: Results Overview: Segmentation

Figure 5 shows an example of the segmented image using different algorithms used in this study. CSF, WM and GM are distinguished by red, yellow and green colors, respectively. Figure 5(a) is slice number 64 from coronal section of image 2 from IBSR dataset. Figure 5(b) is the same image after adding 10 percent bias filed to the image. Figure 5(b) represent the ground truth segmentation. Figure 5(d-h) are the segmentation obtained from image with added bias field by esFCM, FCM, spFCM, sFCM and FCMGsLE, respectively. Focusing on the highlighted area (circle) one can see that the proposed esFCM have a better segmentation with respect to the ground truth.

## Appendix B: Dataset-Specific Findings

### IBSR Dataset

Figures 7 and 6 shows comparisons of the algorithms based on the amount of added noise and bias field in the images, respectively, using criteria such as Hausdorff Distance (HD), DICE, and Accuracy. Both figures highlight better performance of esFCM with respect to the other used algorithms in this study.

Figure 8 shows the specific results for the tissue types with varying amounts of added bias field and noise. It highlights the better perforamnce of the proposed esFCM, specifically for GM and WM segmentation.

### HUPM Dataset

Figures 9 and 10 shows comparisons of the algorithms based on the amount of added noise and bias field in the images, respectively, using the three different criteria. Both figures highlight better performance of esFCM with respect to the other used algorithms in this study.

Figure 11 shows the specific results for the tissue types with varying amounts of added bias field and noise. It highlights the better perforamnce of the proposed esFCM, specifically for GM and WM segmentation.

### BrainWeb Dataset

Figures 12 and 13 present algorithmic comparisons based on the levels of added noise and bias field in the images, respectively, utilizing three distinct criteria. These figures illustrate that the performance of esFCM is on par with other algorithms employed in this study.

In scenarios characterized by high levels of noise or bias fields, there is a noticeable reduction in its performance for tissue segmentation. The specific outcomes for various tissue types under varying degrees of added bias field and noise are detailed in Fig. 14. Notably, the diminished performance of esFCM in the presence of elevated noise or bias fields can be realed to the CSF segmentation. While esFCM’s performance in this specific aspect may not rank as the best, it remains comparable to other algorithms used in the study.

### IXI Dataset

Figure 15 presents a comparison of tissue segmentation among GMM, ANTS, and FSL using box plots on the IXI dataset, where the results of esFCM have been considered as the ground truth. This plot provides insights into how the results of esFCM differ from other methods. According to this figure, the ANTs algorithm exhibits greater variation compared to other methods, yet it demonstrates performance more similar to esFCM than the other algorithms, particularly in terms of DICE and accuracy criteria.

## Footnotes

1 Obtained from http://brain-development.org/ixi-dataset.

## Code Availability

The code will be publicly available at https://github.com/BahramJafrasteh/esFCM and has been implemented inside MELAGE Jafrasteh et al. (2023).
